# Plant-Based Natural Products for the Discovery and Development of Novel Anthelmintics against Nematodes

**DOI:** 10.3390/biom10030426

**Published:** 2020-03-09

**Authors:** Maoxuan Liu, Sujogya Kumar Panda, Walter Luyten

**Affiliations:** Department of Biology, KU Leuven, 3000 Leuven, Belgium; liumaoxuan2008@gmail.com (M.L.); walter.luyten@kuleuven.be (W.L.)

**Keywords:** anthelmintic drugs, *C. elegans*, medicinal plants, synergy, toxicity, veterinary medicine

## Abstract

Intestinal parasitic nematodes infect approximately two billion people worldwide. In the absence of vaccines for human intestinal nematodes, control of infections currently relies mainly on chemotherapy, but resistance is an increasing problem. Thus, there is an urgent need for the discovery and development of new anthelmintic drugs, especially ones with novel mechanisms of action. Medicinal plants hold great promise as a source of effective treatments, including anthelmintic therapy. They have been used traditionally for centuries and are mostly safe (if not, their toxicity is well-known). However, in most medicinal plants the compounds active against nematodes have not been identified thus far. The free-living nematode *C. elegans* was demonstrated to be an excellent model system for the discovery of new anthelmintics and for characterizing their mechanism of action or resistance. The compounds discussed in this review are of botanical origin and were published since 2002. Most of them need further studies of their toxicity, mechanisms and structure-activity relationship to assess more fully their potential as drugs.

## 1. Introduction

Intestinal parasitic nematodes continue to pose problems in human and veterinary medicine [1,2]. In the absence of vaccines for human intestinal nematodes, control of infections currently relies mainly on chemotherapy. However, anthelmintic resistance has been widely reported in livestock, and less in dog [1] or human parasites. Thus, there is an urgent need for the discovery and development of new anthelmintic drugs. Nematodes are the most abundant and ubiquitous multicellular organisms on earth, with an estimated 100,000 to 1,000,000 species (https://www.csiro.au/en/Research/Collections/ANIC/Insect-research/Roundworms-Research). They are organisms with long, thin, unsegmented tube-like bodies, a longitudinal digestive tract and an anterior mouth. Their body wall is composed of an outer non-cellular cuticle, a thin hypodermis and musculature [3,4]. They develop from an egg via four larval stages (L1 through L4) into adults. Each of the four larval stages is ended by a moult in which the cuticle is shed [5]. Their sizes range from <1 mm in length (e.g., *Strongyloides stercoralis*) to 30 cm or more (e.g., *Ascaris*). Over 25,000 nematode species have been described, and more than half of them are parasitic for humans, animals or plants [4].

Parasitic nematodes in humans fall into two broad categories: intestinal nematodes and tissue (blood) nematodes. The distinction between these two categories is based on where the adult stage mainly spends its time (in the intestinal lumen or in body tissues). Intestinal parasitic nematodes, which are the most common and persistent parasitic nematodes in humans, infect approximately two billion people worldwide, especially in developing countries. These intestinal parasitic nematodes mainly include *Ascaris lumbricoides*, *Trichuris trichiura*, *Ancylostoma duodenale*, *Necator americanus*, *Strongyloides stercoralis*, *Enterobius vermicularis* and *Capillaria philippinensis*. The first four species are the most widespread and are normally grouped together as soil-transmitted helminths, since they have a similar life cycle [6,7]. The adults live in the human intestine and produce eggs, which are shed with faeces and embryonate in the soil.

These parasite infections can cause detrimental effects on human growth, nutrition, cognition, school performance, work productivity and pregnancy, which may severely impair the quality of life [5,8]. The majority of intestinal parasitic infections occur in children. A thorough meta-analysis demonstrated that deworming children results in statistically significant improvements in almost all primary outcome measures (height, weight, triceps skin fold and mid-upper arm circumference) and in all secondary outcome measures (e.g., height-for-age, weight-for-age, etc.) [9,10]. Moreover, the infections also indirectly cause a considerable disease burden via impairment of the immune system, leading to increased susceptibility to malaria, HIV/AIDS and tuberculosis [11,12].

In addition, gastro-intestinal nematodes are of major economic importance in livestock (including sheep, goats, cattle, horses and pigs). The annual economic losses caused by parasitic nematodes in livestock run into billions of dollars worldwide [13].

## 2. Anthelmintic Drugs

In the absence of vaccines for human intestinal nematodes, the treatment of nematode infections at present mainly relies on chemotherapy. Despite the severe impact on health caused by intestinal nematodes and their high prevalence in humans, the arsenal of anthelmintic drugs is small. Four main anthelmintic drugs are used for treating human intestinal nematodes: pyrantel pamoate, albendazole, mebendazole and levamisole. Many anthelmintic drugs used in humans were first developed and marketed as veterinary drugs [14]. Albendazole and mebendazole have been chosen for mass drug administration programs and work best for ascariasis and hookworm infections [6,10,15]. In the pharmaceutical industry, the progress of anthelmintic drug discovery and development has been quite slow over the past 40 years, even though some available anthelmintic drugs can show side-effects [13,16]. Tribendimidine has entered human clinical trials in the last four decades (approved in China in 2007) [10]. Recently, a trial of another anthelmintic drug “Emodepside” was successfully completed in healthy volunteers. As a next step, DND*i* plans to run a Phase II “proof-of-concept” clinical trial in DRC and Ghana, investigating the safety and efficacy of the drug in people living with onchocerciasis (https://www.dndi.org/diseases-projects/portfolio/emodepside/). To the best of our knowledge, no other novel drug candidates against human intestinal nematodes are in clinical development at present. This is partly due to the limited financial return from anthelmintic drugs and the high cost of drug development [17,18,19]. The majority of people suffering from intestinal nematode infections live in developing countries, which cannot support a profitable drug market.

Table 1 summarises the anthelmintic drugs for intestinal nematode infections in human and/or veterinary medicine and their mechanism of action. There are more anthelmintic drugs currently used in veterinary medicine than in humans; these include fenbendazole, piperazine, mebendazole, albendazole, morantel, pyrantel, levamisole, ivermectin, moxidectin, monepantel, derquantel and emodepside.

### 2.1. Mechanisms of Action of Anthelmintics

The majority of these drugs target ion channel proteins in the nematode.

Piperazine acts as a weak GABA (4-aminobutyric acid)-mimetic in *Ascaris suum* and causes a flaccid, reversible paralysis of body wall muscles. Single-channel recordings show it to be a low efficacy, partial agonist at GABA-gated chloride channels [20,21].

A number of benzimidazoles like albendazole have been developed for anthelmintic use. Benzimidazoles selectively bind with high affinity to parasite β-tubulin and inhibit microtubule polymerization, which results in the disruption of the cytoskeleton and consequent death of the worm [14].

Levamisole is the pure L-isomer of tetramisole. It is an agonist of nicotinic acetylcholine receptors (nAChRs), causing muscle contractions and spastic paralysis of the worms [22,23]. In addition, levamisole stimulates egg-laying in wild-type *Caenorhabditis elegans* (*C. elegans*) [14].

Morantel is a methyl ester analogue of pyrantel, both of which target the L-subtype nAChR in *Ascaris suum* [14]. Recently, morantel was shown to act as an agonist of the nAChR subtype comprising ACR-26/ACR-27 subunits from *Haemonchus contortus* or *Parascaris equorum,* expressed in *Xenopus laevis* oocytes [24].

Macrocyclic lactones (avermectin, ivermectin, abamectin) are produced by the genus *Streptomyces* [14]. They can elicit a potent and persistent paralysis of nematode pharyngeal and body wall musculature and have broad-spectrum activity against nematodes. They are selective agonists of glutamate-gated chloride channels, which are present only in invertebrates like nematodes and insects [25]. In addition, avermectins also act as antagonists of GABA and nicotinic receptors expressed on somatic muscle cells of parasitic nematodes [14].

Monepantel is the first compound of the class of amino-acetonitrile derivatives developed for the control of parasitic nematodes. The principal target of monepantel in *C. elegans* was suggested to be ACR-23, which belongs to the nematode-specific DEG-3 subfamily of nAChRs [26].

Derquantel is the first commercial member of the spiroindoles. It acts as an antagonist of nAChRs to cause flaccid paralysis of parasites and appears to act preferentially on B-type rather than L-type nAChRs [27]. However, the use of derquantel in forward genetic screens has not yet been reported [20].

Tribendimidine is a symmetrical diamidine derivative of amidantel. It has a broad spectrum of action against parasitic nematodes of humans; it is effective against hookworm, *Strongyloides* and *Ascaris,* but not *Trichuris*. A forward genetic screen for tribendimidine-resistant mutants in *C. elegans* found that these were also resistant to L-subtype nAChR agonists, suggesting a common target for tribendimine and levamisole: the L-type nAChR [10]. However, a more recent study suggested that tribendimidine is not selective for the same receptor subtypes as levamisole and that it is more selective for the B-subtype than the L-subtype of nAChRs in *Ascaris suum* [28].

Emodepside is a cyclooctadepsipeptide that targets the calcium-activated potassium channel (SLO-1) of nematodes [29,30].

### 2.2. Resistance to Anthelmintics

In contrast to human anthelmintic drugs, three new anthelmintic drugs have been commercialized in veterinary medicine over the last couple of years: emodepside, monepantel and derquantel [13]. However, anthelmintic resistance has become widespread in livestock worldwide [31]. The onset of drug resistance development can be quite rapid. For instance, resistance to mebendazole emerged merely three years after its introduction to the market. Moreover, drug resistance to recently commercialized anthelmintic drugs (monepantel and derquantel) has already been described (Table 1). To date, drug resistance to tribendimidine and emodepside has not been reported, perhaps because both are used for humans or companion animals only, where resistance in nematodes is not as apparent compared to farm animals [32].

There are several possible drug resistance mechanisms in nematodes: (i) a reduction in the number of receptors, (ii) a deletion or mutation of amino acid(s) (AA) in the gene encoding the drug target, (iii) the absence of bioactivating enzymes. The detailed underlying resistance mechanisms for each anthelmintic drug class remain to be fully elucidated [14].

Anthelmintic resistance to conventional anthelmintic drugs, and even to some recently commercialized anthelmintic drugs, has developed rapidly, which has influenced the success of conventional anthelmintic drugs for the control of intestinal nematodes in livestock. Resistance against one particular anthelmintic drug is typically accompanied by resistance against other members belonging to the same class (i.e., side-resistance, as opposed to cross- and multidrug-resistance, which refers to resistance against two or multiple drugs belonging to different anthelmintic drug classes) [31]. Anthelmintic resistance has been claimed to occur occasionally also in human intestinal nematodes [35,36], but this is controversial [10,37,38]. It may only be a matter of time before this phenomenon becomes common in helminths of humans [23,39]. Therefore, considering the large number of animals and humans infected by intestinal nematodes, the limited number of available anthelmintic drugs and the emergence of resistance to existing anthelmintic drugs, there is an urgent need for novel anthelmintic drugs against intestinal parasitic nematodes, in particular those with novel mechanisms [14,37].

## 3. *C. elegans*

The most direct route for anthelmintic drug discovery is via whole-organism nematode phenotypic screening [40]. However, the growth of parasitic nematodes involves multiple life stages, many of which are difficult to maintain in the laboratory. Many compounds need to be screened for the discovery of new anthelmintic drugs (the compound collections of major pharmaceutical companies contain millions of synthetic chemicals). Therefore, using parasitic nematodes as a model for anthelmintic discovery is typically expensive, labour-intensive and low-throughput, which is impractical [41,42].

*C. elegans* can be an efficient surrogate of parasitic nematodes for anthelmintic drug discovery. It is a free-living nematode around 1 mm in length as an adult. It has a short life cycle (2–3 weeks) and can be easily maintained in the laboratory at low cost, making it amenable to high-throughput screening. The extensive sequence similarity across the nematode phylum has been demonstrated by comparative genomic studies: *C. elegans* shares almost 13,000 genes (~70%) with various other nematode species [43,44]. *C. elegans* has been extensively proven to be an excellent model of intestinal parasitic nematodes for anthelmintic drug discovery thanks to its similarity to parasitic species [41,45,46]. Notably, almost all the anthelmintic drugs on the market are active against *C. elegans* [20,47]. Moreover, *C. elegans* has played an important role in elucidating the mechanism of action of current anthelmintic drugs, since it is amenable to genetic manipulation and mutagenesis [10,20,48].

The assessment of worm motility is considered to be the current gold standard for measuring drug effectiveness for parasitic nematodes in vitro. Furthermore, the disintegration of the parasite body of deceased nematodes or the ability to interrupt the life cycle of nematodes (egg hatch test, larval development test, larval mortality/motility test, larval migration test) are considered and represent further useful and valid parameters for assessing the in vitro anthelmintic effects of new compounds. In addition, the automated measurement of worm movement in liquid media is well-suited for the readily scorable, phenotypic readout required for high-throughput screening [17,40,45,49].

## 4. Anthelmintic Compounds Derived from Medicinal Plants

Plants have been used in traditional medicine from ancient times. Their use was passed down mostly through oral history based on their efficacy and safety for treating particular ailments and eventually was recorded in herbal classics. Biologically active substances with drug-like properties in medicinal plants are responsible for their medicinal effects [50]. Drug discovery from medicinal plants continues to provide an important source of new drugs and drug leads [51]. There are many medicinal-plant-derived drugs that have been introduced to the market worldwide, such as artemether, galantamine and tiotropium [52]. Notably, the discovery of artemisinin (an antimalarial drug) from *Artemisia annua*, which is used in traditional Chinese medicine, was awarded the Nobel Prize in Physiology or Medicine in 2015 [47]. This prize was shared with the discoverers of ivermectin.

Githiori et al. [53] published a review emphasizing ethnoveterinary plant preparations as livestock de-wormers. They advocated “fostering better interaction between traditional healers and scientists to prevent harmful overexploitation, both of local knowledge and of plant species that may have effects against nematode parasites”. The secondary metabolites in medicinal plants are good sources of anthelmintic drug candidates [54,55]. Some active compounds isolated from medicinal plants have shown anthelmintic activity against intestinal nematodes. Recently, Ndjonka et al. [56] reviewed medicinal plants and natural compounds as anti-*Onchocerca* agents. They used online electronic databases from 1990 to 2017 and found only 13 plants with anti-*Onchocerca* activity. A limitation of this review is its focus on only one species. Romero-Benavides et al. [57] conclude that a lot of plant extracts have shown potential anthelmintic activity, but the number of isolated compounds is much lower. They conclude that further studies are needed on isolating active compounds, as well as preclinical trials to obtain new anthelmintics. We also published a review recently on antiparasitic activity but it deals only with the Asteraceae family, focusing on plant extracts and compounds that can inhibit protozoan parasites such as *Plasmodium*, *Trypanosoma*, *Leishmania* and intestinal worms [54].

Here, we review the discovery of medicinal-plant-derived compounds with activity against intestinal nematodes since 2002. The prior period is well covered by two reviews: “Antiparasitic properties of medicinal plants and other naturally occurring products” [58] and “Phytochemical based strategies for nematode control” [59]. We found that for most medicinal plants traditionally used for treating intestinal nematodes, the active compounds have not yet been identified. The reported anthelmintic activity of natural products from medicinal plants against intestinal parasitic nematodes and *C. elegans* is summarized in Table 2 and Table 3, respectively. They are dealt with separately in the next two subsections, but this does not imply that compounds active on *C. elegans* are inactive on intestinal parasitic nematodes, or vice versa.

### 4.1. Natural Products Active against Intestinal Parasitic Nematodes

**Figure 1:**
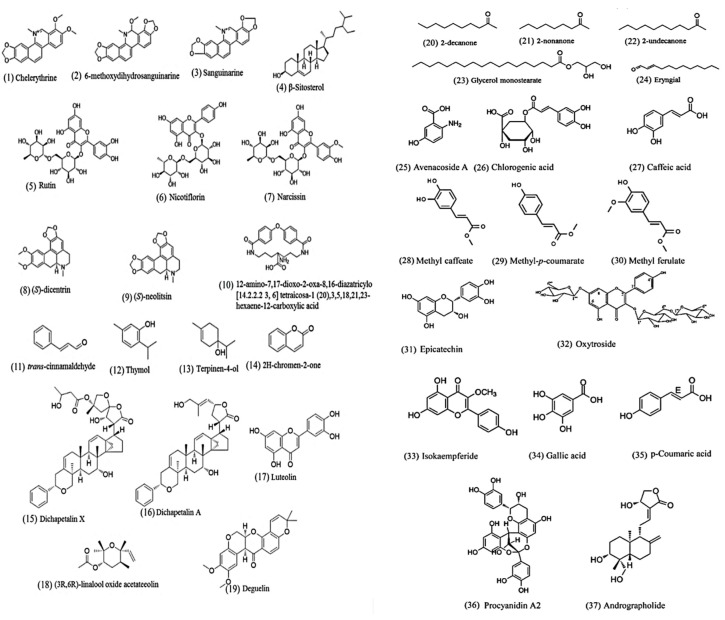
Chemical structures of natural products active against intestinal parasitic nematodes. Lipids (23), phenolics (5,6,7,17,19,26,27,28,31,32,33,34,36), saponin (4,25), terpenoids (12,13,15,16,18,37), alkaloids (1,2,3,8,9), coumaric acid (14,29,30), miscellaneous (10,11,20,21,22,24).

The search term “nematode AND natural product AND anthelmintic" was used to search PubMed (https://www.ncbi.nlm.nih.gov/pubmed/). Only reports on pure compounds isolated from medicinal plants were retained. We found 34 anthelmintic compounds (for their structures, see Figure 1) from medicinal plants active against intestinal parasitic nematodes since 2002. Of these, only eight compounds were evaluated for in vivo anthelmintic activity in animal models (Table 2).

Satou et al. [60] screened several isoquinoline alkaloids on the larvae of *Toxocara canis* (a dog roundworm). Chelerythrine, 6-methoxydihydrosanguinarine and sanguinarine showed the most potent anthelmintic activity. However, these three compounds were highly cytotoxic on HL60 cell lines, with selectivity indexes (the ratio of IC_50_ on worms to CC_50_ on mammalian cells) less than 0.02 [60].

β-Sitosterol isolated from *Mentha cordifolia* showed a similar potency as pyrantel pamoate and mebendazole against *Ascaris suum* in vitro [61]. There are several reports on medicinal plants where β-Sitosterol was found to be the anthelmintic compound against different model organisms [62,63,64,65].

*Onobrychis viciifolia*, a leguminous forage, showed anthelmintic activity, with condensed tannins being considered the main anthelmintic components. In a bioassay-guided fractionation focusing on compounds with molecular weight <2000 Da using a *Haemonchus contortus* larval migration assay, rutin, nicotiflorin and narcissin were identified. Each of these significantly inhibited the migration of L3 worms at 1200 µg/mL [66].

Two aporphine alkaloids, (*S*)-dicentrine and (*S*)-neolitsine, were isolated by a bioassay-guided fractionation from the aerial parts of *Cissampelos capensis*. They exerted a strong anthelmintic activity in a *Haemonchus contortus* larval development assay (EC_90_ = 6.3 and 6.4 μg/mL, respectively). (*S*)-Dicentrine was evaluated for in vivo activity in a mouse model infected by *Heligmosomoides polygyrus*. It showed a 67% reduction of worm counts at an oral dose of 25 mg/kg, compared to >99% for the positive control ivermectin [67].

One new anthelmintic compound was isolated from the stem bark of *Acacia oxyphylla*, which is traditionally used as an anthelmintic in India. Its structure was elucidated as 12-amino-7,17-dioxo-2-oxa-8,16-diazatricylo [14.2.2.2 3, 6] tetraicosa-1 (20),3,5,18,21,23-hexaene-12-carboxylic acid. At 1000 μg/mL, it induced the death of *Ascaridia galli* worms after 15 h [68].

*Eryngium foetidum* is used for food flavouring and for treating intestinal worms in Caribbean folklore. From a bioassay-guided isolation using a *Strongyloides stercoralis* testing model, eryngial (trans-2-dodecenal) was identified as the main anthelmintic compound. Its LD_50_ (461 μM) in a 24 h larval mortality assay is lower than the positive control ivermectin (LD_50_ = 2.25 mM) [69].

Williams et al. [70] found that *Cinnamomum verum* bark extract had anthelmintic activity against *Ascaris suum*. Further phytochemistry analysis revealed that the anthelmintic activity was mainly derived from *trans*-cinnamaldehyde, whose in vivo activity was assessed in a pig model by daily administration (1000 mg/d) in the diet or as a targeted, encapsulated dose (1000 mg, twice daily). However, *Ascaris suum* infection was not significantly decreased. The rapid absorption/metabolism of *trans*-cinnamaldehyde in vivo was proposed as the main reason for this lack of efficacy [70].

Chemical constituents of *Dichapetalum filicaule* were isolated and tested for anthelmintic activity on a *Necator americanus* egg hatch inhibition assay. Three compounds (including a new dichapetalin) were found to be active: dichapetalin X, dichapetalin A and glycerol monostearate [71].

Thymol was demonstrated to be the most important compound for the anthelmintic activity of *Thymus vulgaris* essential oil. It is effective against the three main stages of *Haemonchus contortus*: egg hatching, larval development and adult stages [72].

Terpinen-4-ol from the essential oil of *Melaleuca alternifolia* was shown to possess ovicidal and larvicidal activity against *Haemonchus contortus* [73].

Wangchuk et al. [74] screened four compounds from *Ajania nubigena* on *Trichuris muris* by assessing worm motility using an xCELLigence instrument. Luteolin showed the best activity, while (3R, 6R)-linalool oxide acetate also had good anthelmintic activity. Luteolin was then evaluated in vivo against *Trichuris muris* infection in a mouse model. A single oral dose of 100 mg/kg induced a 27.6% reduction of worm burden, which was much weaker than mebendazole (93.1%) [74].

Dilrukshi Herath et al. screened a natural product library by assessing the motility of L3 larvae of *Haemonchus contortus*. Deguelin (a rotenone derivative) emerged from this screen. It showed a strong anthelmintic activity (IC_50_ = 14.8 μM) and low toxicity against human NFF cells (IC_50_ > 50 μM) [75]. A more recent study suggested that deguelin exerts its anthelmintic activity via the mitochondrial respiratory chain by modulating oxidative phosphorylation [76].

Three compounds, 2-decanone, 2-nonanone and 2-undecanone, from the essential oil of *Ruta chalepensis* demonstrated promising activity against a mixture of sheep gastrointestinal nematodes (*Teladorsagia* spp., *Haemonchus. contortus* and *Trichostrongylus* spp.) [77].

A bioassay-guided fractionation of *Gliricidia sepium* using an egg hatch assay of *Cooperia punctata* led to 2H-chromen-2-one. It inhibited hatching and embryo development with an IC_50_ of 24 µg/mL (164.3 μM) [78].

Avenacoside B, an oat saponin purified from *Avena sativa* green leaves, reduced the infectivity of *Heligmosomoides bakeri* larvae in a mouse model. Avenacoside B induced morphological changes in larvae, enhanced IL-4 production and blocked glycoprotein pump (Pgp) activity [79].

Using bioassay-guided purification from *Tagetes filifolia,* chlorogenic acid proved to be the anthelmintic compound (LC_50_ 248 μg/mL) in an in vitro test (egg hatching or mortality of *H. contortus* larvae) [80].

Castillo-Mitre et al. [81] isolated several caffeoyl and coumaroyl derivatives from *Acacia cochliacantha* with an in vitro egg hatch inhibition test for *H. contortus.* At 1 mg/mL, caffeic acid was most effective (98% inhibition), followed by methyl caffeate and methyl-*p*-coumarate (88%). The fraction containing a mixture of (*p*-coumaric acid + ferulic acid) and (methyl ferulate + quercetin) also showed 94% egg hatch inhibition. The authors concluded that plants from the Leguminosae family may offer an alternative source for the control of gastrointestinal nematodes of small ruminants.

Soldera-Silva et al. [82] hypothesised that avocado seeds may hold promise for anthelmintic applications as they contain polyphenols. They isolated anthelmintic compounds such as epicatechin (EC_50_ = 10 μg/mL) with higher efficacy than rutin (EC_50_ = 30 μg/mL). Additionally, chlorogenic acid was also isolated and tested but did not show significant effects, even though Jasso Diaz et al. [80] previously found (albeit weak) anthelmintic activity.

Wanderley and coworkers evaluated the anthelmintic potential in *H. contortus*-infected sheep of CM-cellulose, a cysteine protease purified from the latex of *Ficus benjamina*. The purified protease inhibited both the development and exsheathment of *H. contortus* larvae, with 50% effective concentrations of 260 and 790 µg/mL, respectively [83].

Kaempferol 3-O-rhamnopyranosyl-(1→6)-β-D-glucopyranoside-7-O-rhamnopyranoside (oxytroside), isolated from *Gliricidia sepium* leaves by bioassay-guided purification, fully inhibited *Cooperia punctata* exsheathment (2400 μg/mL) in calves [84].

Procyanidin A2 (condensed tannin) was isolated from the Australian plant *Alectryon oleifolius* through bioassay-guided purification and demonstrated significant anthelmintic activity in larval development assays, with complete inhibition at 50 μg/mL and an IC_50_ of 12.6 μg/mL [85].

The flavonol isokaempferide was recently isolated from a native Mexican plant (*Baccharis conferta*) and displayed ovicidal effects on *H. contortus* eggs (IC_50_ = 80 µg/mL). From the same plant, the authors also isolated hydroxycinnamic- and 4,5-di-O-caffeoylquinic acid, based on ovicidal effects; however, 100% egg hatching inhibition was only observed at 3 mg/mL [86].

Brazilian red propolis was found to be effective (IC_50_ = 300 µg/mL) in a mouse model infected with *Toxocara cati* [87].

The bioactive molecules (gallic acid and an unidentified compound) from *Caesalpinia coriaria* exhibit in vitro ovicidal activity against several cattle gastrointestinal parasitic nematodes. These galloyl derivatives displayed ovicidal activity of 100% at 1000 µg/mL against *Cooperia* spp., *Haemonchus* spp., *Ostertagia* ssp., *Oesophagostomum* spp. and *Trichostrongylus* spp. [88].

Banerjee et al. [89] isolated andrographolide from an Indian medicinal plant extensively used in Indian traditional medicine for deworming; it showed significant ovicidal and larvicidal activities at 0.125 µg/mL and 19 µg/mL, respectively.

Castaneda-Ramirez et al. [90] recently isolated p-coumaric acid from *Senegalia gaumeri* leaf extract using bioassay-guided purification. They conclude that p-coumaric acid has anthelmintic properties but might act in synergy with other compounds.

### 4.2. Natural Products Active against C. elegans

**Figure 2:**
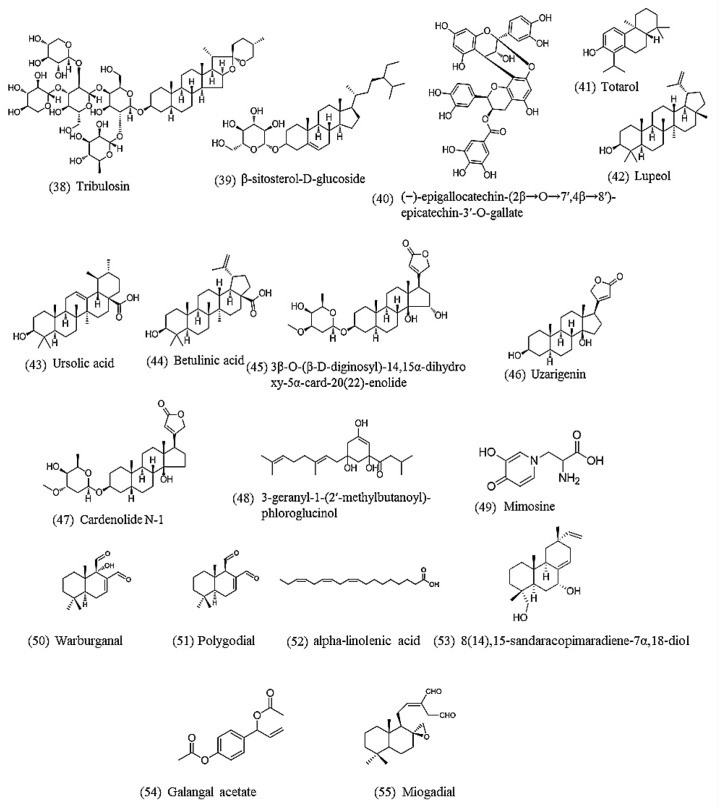
Chemical structures of natural products active against *C. elegans*. Lipids (52), phenolics (40), saponin (38,39), terpenoids (41,42,43,44,49,50,51,53), steroids (45,46,47), miscellaneous (48,54,55).

As in Section 4.1, the term “nematode AND natural product AND anthelmintic" was used for the PubMed search. Only reports on pure compounds active against *C. elegans* isolated from medicinal plants were retained, yielding 18 anthelmintic compounds reported since 2002 (Table 3 and Figure 2).

A bioassay-guided fractionation from *Tribulus terrestris* using *C. elegans* as a test model resulted in two steroidal saponins, tribulosin and β-sitosterol-D-glucoside, that satisfied the activity criterion ED_50_ < 100 μg/mL [65].

Eight compounds were isolated from *Camellia sinensis* and their anthelmintic activity was tested. One new gallate of tannin, (−)-epigallocatechin-(2β → O → 7′,4β → 8′)-epicatechin-3′-O-gallate, showed the best activity with an IC_50_ of 49 μM [91].

A diterpene, totarol, isolated from *Juniperus procera* showed strong nematicidal activity against *C. elegans* at 80 μg/mL (279.3 μM) [92].

Three compounds isolated from a *Curtisia dentata* extract, lupeol, ursolic acid and betulinic acid, were tested on *Haemonchus contortus*, *Trichostrongylus colubriformis* and *C. elegans*. Lupeol and betulinic acid were also active on *Haemonchus contortus* and *Trichostrongylus colubriformis,* but only at high concentrations (200 and 1000 μg/mL, respectively). All three compounds were active against *C. elegans* with an LC_50_ of 2, 12 and 79 μg/mL (4.7, 26.3 and 153.3 μM), respectively [93].

Three anthelmintic cardenolides were isolated from a *Nerium indicum* extract using bioassay-guided purification, one of which was a new compound: 3*β*-*O*-(*β*-D-diginosyl)-14,15*α*-dihydroxy-5*α*-card-20(22)-enolide; the other two were uzarigenin and cardenolide N-1. Their LD_50_ against *C. elegans* after 72 h was 45.4, 177.8 and 41.7 μg/mL (84.9, 474.7 and 80.4 μM), respectively [94].

A phytochemistry study of *Hypericum roeperianum* yielded ten compounds. One of these, 3-geranyl-1-(2′-methylbutanoyl)-phloroglucinol, showed significant anthelmintic activity against *C. elegans*, inducing death in 37% after 30 min treatment at 100 μg/mL (285.3 μM) [95].

Nguyen et al. [96] tested the nematicidal activity of mimosine and its synthetic derivatives in a *C. elegans* model. Mimosine showed the most potent activity with an IC_50_ of 16.8 μM. Structure-activity relationship studies revealed that substituents at the C5-position had a strong impact on the nematicidal activity [96].

Van Puyvelde et al. [97], using bioassay-guided purification, isolated one active compound, 8(14),15-sandaracopimaradiene-7α,18-diol (IC50 = 5.4 ± 0.9 μg/mL), from the leaves of *Tetradenia riparia* using *C. elegans* as a test model. This plant is the most frequently used medicinal plant in traditional Rwandese medicine, and the compound was for the first time reported as having anthelmintic activity.

Liu et al. [98] purified three active compounds by bioassay-guided purification (*C. elegans* motility test) from an African plant *Warburgia ugandensis* Sprague subspecies *ugandensis* (Canellaceae): warburganal (IC_50_ = 28.2 ± 8.6 μM), polygodial (IC_50_ = 13.1 ± 5.3 μM) and alpha-linolenic acid (IC_50_ = 70.1 ± 17.5 μM). A checkerboard assay suggested that warburganal and polygodial both act synergistically with alpha-linolenic acid. A study of the structure-activity relationship for polygodial showed that the α,β-unsaturated 1,4-dialdehyde structural motif is essential for the anthelmintic activity. Additionally, polygodial was equally active against a panel of *C. elegans* mutant strains, resistant against major anthelmintic drug classes, suggesting that polygodial may act via a mechanism that differs from that of currently marketed drugs. The authors demonstrated that polygodial inhibits mitochondrial ATP synthesis of *C. elegans* in a dose-dependent manner (IC_50_ = 1.8 ± 1.0 μM), which is probably the underlying mechanism of action.

The same group also studied in a similar way the anthelmintic activity of the seeds of a well-known traditional Chinese medicinal plant *Torreya grandis* Fortune ex Lindley (Cephalotaxaceae) [99]. Bioassay-guided purification led to two active compounds: galangal acetate and miogadial (IC_50_ = 58.5 ± 8.9 μM and 25.1 ± 5.4 μM, respectively. The two compounds acted synergistically but did not appear to act via TRP channels nor via traditional anthelmintic drug targets.

## 5. Chemistry of Isolated Compounds

Many of the reviewed compounds were isolated using bioassay-guided purification, which offers a better guarantee of identifying (at least the major) bioactive compounds than testing ones isolated in a prior phytochemical study. Although bioassay-guided purification is agnostic about the kind of compound that will be isolated, few truly novel compounds were identified. This may reflect the relative ease of (isolating and) identifying known (vs. novel) natural products.

Many of the reviewed natural products fall into one of several chemical classes, known to comprise anthelmintic compounds.

### 5.1. Lipids

The anthelmintic effects of fatty acids have been reported before [100]. and their activity depends on the chain length as well as the number and position of double bonds [101].

### 5.2. Phenolic Compounds (Including Flavonoids)

Phenolic compounds comprise a broad chemical class, several of whose members show anthelmintic activity (for recent reviews see [102,103]). The activity in phenolic acids increases with the number of hydroxyl groups [101].

### 5.3. Saponins

There are numerous reports of saponins with anthelmintic effects [104,105]. Although most are too toxic for systemic treatment (due to haemolytic effects), their oral administration usually poses no toxicity problems. Therefore, they could be used for intestinal parasitic nematodes.

## 6. Suitability for Drug Development

We are not aware that any of the reviewed compounds are being developed clinically. Presumably, the available information is insufficient to attract industrial interest. One need not look far to discern the reasons.

### 6.1. In Vitro Bioassays

A wide range of bioassays was used (motility, death, egg laying, egg hatching), but there appears to be no consensus on which of these has the best predictive value for successful clinical development. Most current anthelmintics cause motor paralysis, perhaps explaining why motility tests are most popular. However, if we want to discover anthelmintics with novel mechanisms of action, perhaps bioassays should be broadened to other phenotypes, like egg laying or hatching [106]. It is, however, not clear by how much these parameters need to be reduced in order to obtain clinical efficacy [107].

Although various life stages were used in different studies, the L3 stage appears to be most popular, especially in *C. elegans* [20].

It is often not reported whether the bioactive compound exerts a transient or permanent (lethal) effect. The latter appears preferable on theoretical grounds, but some clinically useful anthelmintics (like levamisole) produce a transient motor paralysis [20].

### 6.2. Potency

The potency of the reported compounds spans at least three orders of magnitude, from >1000 µg/mL to around 1 µg/mL (Table 2 and Table 3). In cases where activity is only seen in the mM range, one should be reticent to conclude that these constitute the bioactive compounds from the plant in question, especially if they were not obtained using bioassay-guided purification. Of course, differences in bioassay type and measured parameters, as well as species differences, may contribute to this wide distribution. However, even in one species (*C. elegans*), the values span a wide range. This range is similar to that for active compounds from a chemical library [44] and overlaps in its lower range with IC_50_ values for some clinically used anthelmintics. This is perhaps not surprising since many of the compounds were purified from plants traditionally used to treat intestinal worms. Nonetheless, further improvement in relevant characteristics of these natural products can most likely be obtained by (semi)synthesis of analogues. This may also permit filing composition of matter patents, which in turn would increase the commercial attractiveness. Analogue (semi)synthesis may be out of the reach of most academic parasitology labs, although collaboration with an organic synthesis group that has experience with the relevant class of natural products may be feasible [108]. In the meantime, testing commercially available analogues may already provide a first glimpse of the structure-activity relationship, which should be highly useful for subsequent analogue design.

### 6.3. Synergy

In a few cases, synergy was reported between bioactive compounds isolated from the same plant [98,99,109]. Such synergies appear common in medicinal plants and may explain the relatively low potency of individual phytoconstituents [110]. They may offer advantages like lower risk of resistance against the crude botanical preparation, especially if their mechanisms differ. Synergy with clinically used anthelmintics was rarely tested [111]. This is unfortunate since potentially useful combination therapies of natural products with synthetic anthelmintics could thus be found [112]. These could in principle be patented and might overcome resistance [113]. It would therefore be useful to test more systematically for such synergies.

### 6.4. Spectrum

Most studies use only a single target organism, which in many cases is not a parasite (i.e., *C. elegans*). In vitro evidence of activity on a suitable parasite is probably essential for progressing a compound for further development, although well-documented anthelmintic traditional use, in conjunction with *C. elegans* activity, gives more confidence that the activity will persist on parasites, compared to compounds emerging from a screen of a chemical library [114,115].

Most clinical anthelmintics act on a wide range of intestinal nematodes. Such broad spectrum is required for situations where diagnosis of the causative parasite(s) is impractical, such as for livestock (where mixed infections are common) or in developing countries (where diagnostic resources are limited). To become attractive drug development candidates, activity of the natural product on most of the common parasites will presumably have to be demonstrated [111].

### 6.5. Toxicity

Very few studies report toxicity measures for the anthelmintic compounds [116]. These are nonetheless essential for assessing their clinical development potential. Toxicity tests on mammalian cells are popular and fairly easy, but these do not predict in vivo toxicity well [117]. Moreover, cytotoxicity may not be very relevant for compounds that stay in the gastrointestinal lumen.

### 6.6. Pharmacokinetics

Only in one reviewed publication was the fate of the active compound studied, perhaps because of the lack of an in vivo effect, notwithstanding solid in vitro activity. Undertaking a pharmacokinetic study is difficult [118], particularly for most academic labs. It may also appear somewhat superfluous at early stages if good activity in vivo can be demonstrated. The gastrointestinal tract is a small compartment, and high concentrations can be reached with limited amounts of active compound, provided it is not absorbed or broken down [119]. The former is of course highly undesirable for tissue nematodes. It is clear that the development criteria for anthelmintics to combat intestinal or tissue nematodes will be quite different.

### 6.7. In Vivo Effects

Only a few studies also provide data on in vivo efficacy and most use a lab animal model. These tests are significantly more cumbersome and expensive [120], but of course ultimately necessary for progression to clinical development. Since most academic labs have no easy access to them, this remains a major hurdle.

### 6.8. Mechanism of Action

Few studies determine the mechanism of action of their anthelmintic compound [98,99,101,121]. This is understandable given the difficulty of such an undertaking and the amount of effort involved. Nonetheless, testing the compound on a panel of *C. elegans* mutants, each resistant to a known anthelmintic class, could quickly identify the compounds with a presumptive novel mechanism of action [99].

Several natural products appear to exert their anthelmintic activity via mitochondrial inhibition, without undue toxicity for the host [122].

## 7. Conclusions

From this brief overview, it is evident that over the past 15 years over three dozen anthelmintic compounds were isolated from medicinal plants, most of which are used traditionally to treat gastrointestinal nematodes. Most of these compounds were found to be effective in in vitro tests (in over half of the cases on a parasitic nematode), but few were examined in an in vivo model. If further (pre)clinical development of these compounds is desired, additional evidence will have to be collected. Academic labs are typically not well-positioned to undertake many of these additional studies, although testing commercially available analogues to get an initial idea of the structure-activity relationship, cytotoxicity and activity on a panel or resistant mutants should be within their reach. For additional toxicity studies, pharmacokinetics and chemical synthesis, academic drug discovery platforms (http://addconsortium.org/about-landing.php) can be approached. We hope that this review will encourage basic scientists to study the mechanism of action of anthelmintic compounds using *C. elegans* as a model organism. Only with these further studies will bioactive compounds from traditional medicine become sufficiently attractive for pharmaceutical or biotech companies, which will probably be necessary to develop them further into novel anthelmintic drugs.

## Figures and Tables

**Table 1:** Anthelmintic drugs for intestinal nematode infections in human and/or veterinary medicine (modified from [31]).

Drug Class	Mechanism of Action	Drug Name	Year of Initial Approval	Year of First Resistance Report
Heterocyclic compounds	Agonist of the inhibitory GABA-receptor	Piperazine	1954	1966
Benzimidazoles	Inhibiting microtubule polymerisation	Mebendazole	1972	1975
		Albendazole	1972	1983
Tetrahydropyri-midines	Agonist of nicotinic acetyl-choline receptors	Morantel	1970	1979
	Agonist of nicotinic acetyl-choline receptors	Pyrantel	1974	1996
Imidazothiazoles	Agonist of nicotinic acetyl-choline receptors	Levamisole	1970	1979
Macrocyclic lactones	Allosteric modulators of glutamate-gated chloride channels	Ivermectin	1981	1988
		Moxidectin	1991	1995
Amino-acetonitrile derivatives	Agonist of nicotinic acetyl-choline receptors	Monepantel	2009	2013 [33]
Spiroindole	Antagonist of nicotinic acetyl-choline receptors	Derquantel	2010	2016 [34]
Aminophenylamidines	Agonist of nicotinic acetylcholine receptors	Tribendimidine ^a^	2004	-
Cyclooctadepsipeptide	Activating a SLO-1-depen-dent pathway	Emodepside ^b^	2005	-

^a^: for humans only, ^b^: for cats and dogs only.

**Table 2:** List of natural products derived from medicinal plants against intestinal parasitic nematodes.

Active Compounds	Plant	Parasite Model	Anthelmintic Activity			Reference
			In Vitro	In Vivo	Assay	
Chelerythrine	*Chelidonium majus*	*Toxocara canis*	IC_50_ = 28 μΜ	nd	Mortality after 24 h	[60]
6-Methoxydihydrosanguinarine	*Macleaya cordata*		IC_50_ = 18 μΜ	nd		
Sanguinarine	*Macleaya cordata*		IC_50_ = 58 μΜ	nd		
β-Sitosterol	*Mentha cordifolia*	*Ascaris suum*	60 mM induced paralysis of worm in 1 h	nd	Paralysis	[61]
Rutin	*Onobrychis viciifolia*	*Haemonchus contortus*	Migration was reduced by 25% at 1965 μM	nd	Larval migration inhibition for 3 h	[66]
Nicotiflorin			Migration was reduced by 30% at 2018 μM	nd		
Narcissin			Migration was reduced by 35% at 1921 μM	nd		
(*S*)-Dicentrine	*Cissampelos capensis*	*H. contortus*	EC_90_ = 6.3 μg/mL (18.5 μM)) in a larval development assay	25 mg/kg dosed orally resulted in 67 % reduction of worm counts in a mouse model infected by *Heligmosomoides polygyrus*	Larval development in vitro/ worm counts in vivo	[67]
63(*S*)-Neolitsine			EC_90_ = 6.4 μg/mL (19.8 μM) in a larval development assay	nd		
12-Amino-7,17-dioxo-2-oxa-8,16-diazatricylo [14.2.2.2 3, 6] tetraicosa-1 (20),3,5,18,21,23-hexaene-12-carboxylic acid	*Acacia oxyphylla*	*Ascaridia galli*	50, 100 and 1000 μg/mL (121, 242 and 2420 μM) induced the death of worms after 30 h, 22 h and 15 h.	nd	Mortality	[68]
Eryngial	*Eryngium foetidum*	*Strongyloides stercoralis*	LD_50_ = 461 μM	nd	Larval mortality after 24 h	[69]
*trans*-Cinnamaldehyde	*Cinnamomum verum*	*A. suum*	25.6 μg/mL (193.8 μM) induced larval death within 3 h	Infection was not signify-cantly decreased by daily administration in the diet (1000 mg/d) or as a targeted, encapsulated dose (1000 mg, twice daily) in a pig model	Larval mortality after 12 h in vitro/ larval burden in vivo	[70]
Dichapetalin X	*Dichapetalum filicaule*	*Necator americanus*	IC_50_ = 744.4 μM	nd	Egg hatch inhibition assay	[71]
Dichapetalin A			IC_50_ = 277.7 μM			
Glycerol monostearate			IC_50_ = 853.4 μM			
Thymol	*Thymus vulgaris*	*H. contortus*	Effective against the three main stages of parasites: IC_50_ = 2.9 mM against egg hatching; IC_50_ = 3.3 mM against larval motility; 16.6 mM completely inhibited the movement of adult worms within 8 h	nd	Egg hatching; motility of worms	[72]
Terpinen-4-ol	*Melaleuca alternifolia*	*H. contortus*	LC_50_ = 4.1 mM, LC_90_ = 20.2 mM in egg hatching assay; 22.7 mM induced a 82.4% inhibition of larval migration	nd	Egg hatching; inhibition of larval migration	[73]
Luteolin	*Ajania nubigena*	*Trichuris muris*	IC_50_ = 9.7 μg/mL (33.9 μM)	A single oral dose of 100 mg/kg induced a 27.6% reduction of worm burden in a mouse model	Mortality of adult worms after 12 h in vitro/ worm burden in vivo	[74]
(3R,6R)-Linalool oxide acetate			IC_50_ = 20.4 μg/mL (96.1 μM)	nd		
Deguelin	*Mundulea sericea*	*H. contortus*	IC_50_ = 14.8 μM	nd	Larval mortality after 72 h	[75,76]
2-Decanone	*Ruta chalepensis*	*Teladorsagia* spp. (52%)*, Haemonchus. contortus* (25%) *and Trichostrongylus* spp. (23%)	IC_50_ = 447.9 μM	nd	Immotile/paralysis after 24 h	[77]
2-Nonanone			IC_50_ = 1757.5 μM	nd		
2-Undecanone			IC_50_ = 5167.5 μM	Nd		
2H-Chromen-2-one	*Gliricidia sepium*	*Cooperia punctata*	IC_50_ = 164.3 μM	nd	Egg hatch inhibition assay	[78]
Avenacoside	*Avena sativa*	*Heligmosomoides bakeri*	Avenacosides change the molecular pattern of nematode larva proteins and block glycoprotein pump activity.	Mouse model	Larval development assay	[79]
Chlorogenic acid	*Tagetes filifolia*	*H. contortus*	LC_50_ 248 μg/mL	nd	Egg hatch inhibition assay	[80]
Caffeoyl and coumaroyl derivatives	*Acacia cochliacantha*	*H. contortus*	With concentration 1 mg/mL several compounds show egg hatch inhibition: caffeic acid (98%), methyl caffeate (88%), methyl-*p*-coumarate (88%) and methylferulate (75%). Additionally, *p*-coumaric acid and ferulic acid mixture and methyl ferulate and quercetin also showed 94% egg hatch inhibition.	nd	Egg hatch inhibition assay	[81]
Epicatechin, rutin	*Persea americana*	*H. contortus*	Epicatechin (EC_50_ = 10 μg/mL), rutin (EC_50_ = 30 μg/mL)	Goat	Larval migration inhibition assay	[82]
CM-cellulose, a cysteine protease	*Ficus benjamina*	*H. contortus*	EC_50_ value for larval development = 0.22 mg/mL, EC_50_ value for larval exsheathment = 0.79 mg/mL	Sheep	Larval development and exsheathment inhibition assay	[83]
Kaempferol 3-O-rhamnopyranosyl-(1 → 6)-β-D-glucopyranoside-7-O-rhamnopyranoside	*Gliricidia sepium*	*C. punctata*	Fully inhibited the *C. punctata* exsheathment process at 2400 µg/mL in calves	Calves	Larval development and exsheathment inhibition assay	[84]
Procyanidin A2	*Alectryon oleifolius*	*cyathostomins*	IC_50_ = 12.6 μg/mL	nd	Larval migration inhibition assay	[85]
Isokaempferide	*Baccharis conferta*	*H. contortus*	IC_50_ = 80 µg/mL	nd	Egg hatching inhibition assay	[86]
EO	Brazilian Red Propolis	*Toxocara cati*	IC_50_ = 300 μg/mL	In mouse model, at 600 μg/mL after exposure for 48 h, shows larvicidal activity	Larval mortality after 48h (in vitro and in vivo)	[87]
Gallic acid	*Caesalpinia coriaria*	Gastrointestinal nematodes (*Cooperia* spp, *Haemonchus* spp., *Ostertagia* ssp., *Trichostrongylus* spp. and *Oesophagostomum* spp.)	The bioactive molecules (gallic acid and unidentified compound) displayed an ovicidal activity of 100% at 1000 µg/mL.	nd	Egg hatching inhibition assay	[88]
Andrographolide	*Andrographis paniculata*	*Ancylostoma duodenale*	Andrographolide exhibits significant ovicidal and larvicidal activity at 0.125 µg/mL and 19 µg/mL, respectively.	nd	Egg hatching inhibition assay	[89]
p-Coumaric acid	*Senegalia gaumeri*	*H. contortus*	At 400 μg/mL ovicidal effect of 8.7%, a larvae failing eclosion effect of 2.9%, and 88.4% of the emerged larvae were damaged.	nd	Egg hatching inhibition assay	[90]

**Table 3:** List of natural products derived from medicinal plants active against *C. elegans*.

Active Compounds	Plant	Anthelmintic Activity			Reference
		In Vitro	In Vivo	Assay	
Tribulosin	*Tribulus terrestris*	ED_50_ = 66.0 μM	nd	Immotile/paralysis after 18 h	[65]
β-Sitosterol-D-glucoside		ED_50_ = 142.1 μM	nd		
(−)-Epigallocatechin-(2β→O→7′,4β→8′)-epicatechin-3′-O-gallate	*Camellia sinensis*	LC_50_ = 49 μM	nd	Mortality after 96 h	[91]
Totarol	*Juniperus procera*	279.3 μM showed strong nematicidal activity	nd	Mortality after 24 h	[92]
Lupeol	*Curtisia dentata*	LC_50_ = 4.7 μM	nd	Immotile/paralysis after 7 d	[93]
Ursolic acid		LC_50_ = 26.3 μM	nd		
Betulinic acid		LC_50_ = 153.3 μM	nd		
3*β*-*O*-(*β*-D-Diginosyl)-14,15*α*-dihydroxy-5*α*-card-20(22)-enolide	*Nerium indicum*	LC_50_ = 84.9 μM	nd	Mortality after 72 h	[94]
Uzarigenin		LC_50_ = 474.7 μM	nd		
Cardenolide N-1		LC_50_ = 80.4 μM	nd		
3-Geranyl-1-(2′-methylbutanoyl)-phloroglucinol	*Hypericum roeperianum*	100 μg/mL (285.3 μM) induced a death percentage of 37%	nd	Mortality after 30 min	[95]
Mimosine	*Leucaena leucocephala*	IC_50_ = 16.8 μM	nd	Mortality after 48 h	[96]
(14),15-Sandaracopimaradiene -7α,18-diol	*Tetradenia riparia*	IC_50_ = 5.4 ± 0.9 µg/mL (17.8 ± 2.9 µM).	nd	Motility test using WMicrotracker,	[97]
Warburganal, polygodial, alpha-linolenic acid	*Warburgia ugandensis*	Warburganal (IC_50_: 28.2 ± 8.6 μM), polygodial (IC_50_: 13.1 ± 5.3 μM) and α-linolenic acid (IC_50_: 70.1 ± 17.5 μM)	nd	Motility test using WMicrotracker,	[98]
Galangal acetate, miogadial	*Semen torreyae*	Galangal acetate (IC_50_: 58.5 ± 8.9 μM) and miogadial (IC_50_: 25.1 ± 5.4 μM)	nd	Motility test using WMicrotracker,	[99]
